# Efficacy and Safety of Conversion Therapy by Intraperitoneal and Intravenous Paclitaxel Plus Oral S-1 in Gastric Cancer Patients With Peritoneal Metastasis: A Prospective Phase II Study

**DOI:** 10.3389/fonc.2022.905922

**Published:** 2022-06-20

**Authors:** Zhong-Yin Yang, Fei Yuan, Sheng Lu, Wei Xu, Jun-Wei Wu, Wen-Qi Xi, Min Shi, Zhen-Qiang Wang, Zhen-Tian Ni, Chang-Yu He, Xue-Xin Yao, Ya-Nan Zheng, Zheng-Lun Zhu, Wen-Tao Liu, Jun Zhang, Huan Zhang, Chen Li, Chao Yan, Min Yan, Zheng-Gang Zhu

**Affiliations:** ^1^ Department of General Surgery, Gastrointestinal Surgery, Shanghai Key laboratory of Gastric Neoplasms, Shanghai Institute of Digestive Surgery, Ruijin Hospital, Shanghai Jiao Tong University School of Medicine, Shanghai, China; ^2^ Department of Pathology, Ruijin Hospital, Shanghai Jiao Tong University School of Medicine, Shanghai, China; ^3^ Department of Oncology, Ruijin Hospital, Shanghai Jiao Tong University School of Medicine, Shanghai, China; ^4^ Department of Radiology, Ruijin Hospital, Shanghai Jiao Tong University School of Medicine, Shanghai, China

**Keywords:** gastric cancer, peritoneal metastasis, intraperitoneal chemotherapy, paclitaxel, conversion surgery

## Abstract

**Background:**

Neoadjuvant intraperitoneal and systemic chemotherapy (NIPS) has shown promising results in gastric cancer (GC) with peritoneal metastasis. However, clinical practice experience of NIPS is still lacking in China. In this study, we investigate the efficacy and safety of NIPS in Chinese patients.

**Methods:**

Eligible patients received NIPS every 3 weeks. Gastrectomy was performed for patients who met the criteria of conversion surgery. The primary end point was 1-year overall survival (OS) rate. Secondary end points were the response rate, toxic effects, conversion surgery outcomes and median survival time (MST).

**Results:**

Sixty-seven patients were enrolled. The primary endpoint was achieved with 1-year OS rate reached 67.2% (95% CI, 56.8%-79.4%). Conversion surgery was performed in 42 patients (62.9%), and R0 resection was achieved in 23 patients (54.8%) with the MST of 31.3 months (95% CI, 24.3-38.3). And the MST was 19.3 months (95% CI, 16.4-22.2) for all patients. Toxicity and surgical complications were well-tolerated. Moreover, sex, R0 resection, pathological nodal stage and tumor regression grade (TRG) were independent prognostic factors for patients who underwent conversion surgery.

**Conclusion:**

The NIPS is effective and safe in treating GC patients with peritoneal metastasis. Male patients, patients who underwent R0 resection, patients with ypN0-1 or TRG 1 after conversion surgery are more likely to benefit from the NIPS.

**Clinical Trial Registration:**

http://www.chictr.org.cn/, identifier https://clinicaltrials.gov/ (<ChiCTR2200056029>).

## Introduction

Gastric cancer is among the most common cancers with poor prognosis worldwide (1). In particular, GC is the fifth most commonly diagnosed cancer and the third leading cause of death in China, accounting for 44.1% and 49.9% of the cases worldwide (2). Peritoneal metastasis remains the most frequent mode of metastasis and is the main cause of mortality in GC (3). Despite treatment with platinum and fluorouracil based systemic chemotherapy, patients with peritoneal metastasis still have a poor survival time of merely 6 to 15 months (4, 5). Cytoreductive surgery (CRS) and hyperthermic intraperitoneal chemotherapy (HIPEC) were reported efficacious in specialized centers with a median survival of 11.0 months (6). However, the morbidity and mortality of CRS/HIPEC deterred the wildly using of the treatments (7).

In China, about 70% of the new diagnosed cases with GC are in the advanced stage, and many of them had peritoneal metastasis (8). However, there are still no generally accepted chemotherapy regimens and also no prospective studies on systemic chemotherapy combined with intraperitoneal chemotherapy for the treatment of these patients in China. With regard to this situation, we conducted the DRAGON series of clinical trials, and DRAGON-01 includes the phase II and phase III clinical trials on neoadjuvant intraperitoneal and systemic chemotherapy (NIPS) for GC with peritoneal metastasis. The NIPS contained weekly intravenous and intraperitoneal paclitaxel (PTX) plus oral tegafur-gimeracil-oteracil potassium capsules (S-1). And we performed conversion surgery for patients with disappearance or remarkable shrinkage of the peritoneal metastasis. In the current study, we evaluated the efficacy and safety of the NIPS therapy and to explore how the outcome of NIPS treatment can lead to a survival benefit for patients.

## Materials and Methods

### Study Design and Participants

This single-group, phase II trial was undertaken in Ruijin Hospital Shanghai Jiao Tong University School of Medicine. The concise eligibility criteria were as follows: histologically confirmed gastric adenocarcinoma, peritoneal metastases from gastric cancer requiring definitive diagnosis by laparoscopy not just positive peritoneal cytology (female patients with ovarian metastases were eligible), without gastric outflow tract obstruction and intestinal obstruction, no prior treatment with chemotherapy, radiation therapy, targeted therapy or immunotherapy, age between 18 and 75 years, Eastern Cooperative Oncology Group (ECOG) score ≤ 2, expected life expectancy ≥ 3 months, and adequate organ function. Detailed inclusion and exclusion criteria are listed in **Supplementary Table S1**. This study was conducted with the approval of the Ruijin Hospital Ethical Review Board. All patients provided written informed consents.

### Treatment

Patients diagnosed with peritoneal metastasis were confirmed by laparoscopy, and intraperitoneal ports were implanted in the subcutaneous space of the lower abdomen. Ascites or lavage fluid was gathered at the first- and the second-look laparoscopy and sent for laboratory examination. The extent of peritoneal metastasis was classified according to the Japanese classification of gastric carcinoma 12th edition and 1st English edition (9), and the peritoneal cancer index (PCI) score was calculated and recorded at the time of laparoscopy (10).

The regimen consisted of intravenous PTX 50 mg/m^2^, intraperitoneal PTX 20 mg/m^2^ on days 1 and 8 and plus oral S-1 at a dose of 80 mg/m^2^ on days 1-14, as reported previously (11). The regimen was repeated every 3 weeks until disease progression or intolerable toxicity.

The criteria for the second-look laparoscopy was set as follows: 1) disappearance or remarkable shrinkage of peritoneal metastasis by imageological examination; 2) negative peritoneal cytology; 3) no other distant metastasis; 4) downstaging of the primary tumor; 5) the patient’s general condition improved. The same combination chemotherapy regimen was given as soon as possible after gastrectomy, and appropriate dose reduction was recommended if the patients could not tolerate the preoperative dosage.

### Assessments

The amount of ascites was evaluated by CT scan and categorized as none, small (within the pelvic cavity) or moderate (beyond the pelvic cavity) at enrollment.

To estimate the response to chemotherapy, patients underwent a second CT scan and maximum tumor area change was assessed in accordance to the WHO criteria (12). The radiological response was graded into complete response (CR), partial response (PR), stable disease (SD) and progressive disease (PD). CR and PR were regarded as objective responses, and CR, PR and SD were regarded as disease controls. Pathological response was defined as residual tumor cells with tumor regression grade (TRG) less than grade 3 (13). Toxicity was evaluated and graded according to the National Cancer Institute Common Terminology Criteria for Adverse Events (version 4.0). Surgical safety was assessed by the incidence of surgical and port related complications based on the Clavien-Dindo classification (14). Pathological response to NIPS after conversion surgery was evaluated based on TRG by the Becker criteria (15).

### Statistics

Based on a phase 3 clinical trial in unresectable or recurrent GC, the threshold set for the 1-year OS rate was determined to be 54% (16). The expected 1-year OS rate was set at 65% based on previous reports (17, 18). With a 2-sided, type I error of 0.05, power of 0.8, and follow-up of 3 years after the closure of recruitment, the enrollment of 67 patients was necessary.

Descriptive statistics of clinicopathological characteristics were performed. The 95% confidence intervals (CIs) of the median survival time were calculated. We used the Kaplan-Meier method to estimate survival curves. Univariate and multivariate Cox regression analyses were used to evaluate the prognostic significance of clinicopathological factors. *P*<0.05 was considered statistically significant. Analyses were performed using SPSS version 26.0.

## Results

### Patients Clinical Characteristics

Between May 2015 and September 2017, 67 patients with a median (interquartile range [IQR]) age of 51 (25–76) years were enrolled in this study. Follow-up was continued for 3 years. The baseline clinical characteristics of patients before NIPS therapy were listed in **Table 1**. All patients received at least 3 courses of NIPS therapy, 45 patients (67.2%) met the criteria of the second-look exploration and 42 (62.7%) proceeded with the conversion surgery. Most of the patients had an Eastern Cooperative Oncology Group performance status (ECOG PS) of 0 or 1 (87.8%). In particular, patients with P2/P3 peritoneal metastasis accounted for the majority of all patients (97%), 86.6% (58/67) of patients had small to moderate degrees of ascites, and 89.6% (60/67) of patients with PCI scores ≥ 10. In female patients, 59.1% (26/44) had ovarian metastasis accompanied by peritoneal metastasis.

**Table 1:** Baseline characteristics.

Variables	n	%
Sex		
Male	23	34.3
Female	44	65.7
Age		
< 60	51	76.1
≥ 60	16	23.9
BMI		
< 23	44	65.7
≥ 23	23	34.3
ECOG PS		
0	27	41.5
1	31	46.3
2	9	12.2
Peritoneal metastasis		
P1	2	3.0
P2	5	7.5
P3	60	89.5
Amount of ascites		
None	9	13.4
Small	26	38.8
Moderate	32	47.8
Histologic type		
Adenocarcinoma	53	79.1
Mucinous cell	3	4.5
Signet ring cell	11	16.4
PCI score		
0-9	7	10.4
10-19	30	44.8
20-39	30	44.8
Pathological grading		
Moderately	2	3.0
Poorly	42	62.7
Unknown	23	34.3
Ovarian metastasis (Female)		
With	26	59.1
Without	18	40.9

### Adverse Events

During NIPS treatment, the most common grade 3 and grade 4 toxic effects were leukopenia (19.4%), neutropenia (26.9%) and anemia (31.3%). Most of the hematological and nonhematological adverse events were below grade 3 (**Table 2**). Intraperitoneal port related complications were observed in 17 patients (25.3%). Subcutaneous liquid accumulation (8/17, 47.1%) and infection (4/17, 23.5%) were the main complications. However, most complications were grade 1 and grade 2 and were controlled through conservative treatments. Grade 4 port-related complications were remedied by replacing new ports, and peritoneal infusion could be continued. There were no treatment-related deaths or unexpected serious adverse events (**Supplementary Table S2**).

**Table 2:** Treatment-related adverse events.

Toxicity	Grade				
	1	2	3	4	3/4 (%)
Leukopenia	16	22	9	4	19.4
Neutropenia	12	19	14	4	26.9
Anemia	18	16	15	6	31.3
Thrombocytopaenia	4	4	4	0	6.0
ALT increased	15	7	3	0	4.5
AST increased	19	6	3	0	4.5
Creatinine increased	4	3	0	0	0
Febrile neutropenia	5	4	2	0	3.0
Fatigue	22	8	0	0	0
Nausea	12	4	1	0	1.5
Vomiting	6	2	0	0	0
Diarrhea	7	2	1	0	1.5
Anorexia	17	3	2	0	3.0
Peripheral neuropathy	11	3	0	0	0
Rash	5	0	0	0	0
Oral mucositis	12	1	0	0	0
Abdominal pain	10	6	2	0	3.0
Alopecia	25	8	5	0	7.5

AST, Aspartate transaminase; ALT, alanine transaminase. NCI-CTCAE version 4.0.

### Outcomes of Conversion Surgery

After a median of 6 courses (range, 3-12 courses) of NIPS therapy, 45 patients with negative peritoneal cytology showed a clinical response, and second-look laparoscopic exploration was performed. The disappearance or remarkable shrinkage of peritoneal metastasis was observed in 42 patients who proceeded to undergo the conversion surgery.

Thirty patients underwent total gastrectomy, and 12 patients underwent distal gastrectomy. D2 lymphadenectomy was performed in 38 patients and D1 lymphadenectomy in 4 patients. R0 resection was performed in 23 patients, while there was no R1 resection; however, in 19 patients, who showed remarkable shrinkage pf peritoneal metastasis, some metastatic nodules were difficult to resect during the surgery (R2). Adnexectomy was performed in 14 patients with ovarian metastasis. Combined gastrectomy with distal pancreatectomy and splenectomy were performed in 2 patients due to tumors invading the tail of the pancreas, and cholecystectomy was performed in 1 patient. The median number of resected metastatic lymph nodes was 6 (range, 0 - 32), and the numbers of patients with ypN0, ypN1, ypN2, ypN3a and ypN3b were 11, 4, 8, 13 and 6, respectively (**Table 3**).

**Table 3:** Clinicopathological characteristics of patients with conversion surgery.

Variables	n	%
Sex		
Male	12	28.6
Female	30	71.4
Preoperative NIPS courses		
< 6	21	50.0
≥ 6	21	50.0
Type of gastrectomy		
Distal	12	28.6
Total	30	71.4
Extent of resection		
R0	23	54.8
R2	19	45.2
Combination of resection		
Ovary	14	33.3
Gall bladder	1	2.4
Distal pancreas and spleen	2	4.8
Lymphadenectomy		
D1	4	9.5
D2	38	90.5
Primary tumor location		
Proximal	4	9.5
Body	26	61.9
Distal	10	23.8
Whole	2	4.8
Pathological tumor stage (ypT)		
ypT0	5	11.9
ypT1	1	2.4
ypT2	4	9.5
ypT3	8	19.0
ypT4a	21	50.0
ypT4b	3	7.1
Pathological nodal stage (ypN)		
ypN0	11	26.2
ypN1	4	9.5
ypN2	8	19.0
ypN3a	13	31.0
ypN3b	6	14.3
Metastatic ovary resection (Female, n=18)		
Yes	14	77.8
No	4	22.2

Surgery-related complications were observed in 18 patients. Ileus was the most frequent complication, occurring in 5 patients, and other complications, including intra-abdominal bleeding, pancreatic fistula, wound infection, anastomotic leakage, abdominal infection, pulmonary infection, sepsis, stenocardia and perineum edema (**Supplementary Table S3**). There were no surgery-related deaths.

### Tumor Responses and Histopathological Tumor Regression

Of the 67 patients, 64 had image-based measurable disease, including 22 who showed PR, 39 who showed SD and 3 who showed PD in accordance to WHO criteria. Therefore, the overall response rate (ORR) was 34.4%, and the disease control rate (DCR) (PR plus SD) was 95.3%. Forty-seven of 67 (70.1%) patients had positive cytology, and peritoneal cytology turned negative in 39 (82.9%) patients. The overall pathological response (TRG grade 1a + 1b + 2) was 71.4% (30/42), and 33.3% (14/42) of the patients achieved complete and subtotal regression (TRG 1a+1b) (**Table 4**).

**Table 4:** Tumor responses and histopathological tumor regression.

Response	n	%
WHO Criteria (n=64)		
Complete response	0	0
Partial response	22	34.4
Stable disease	39	60.9
Progressive disease	3	4.7
Peritoneal cytology positive	47	70.1
Cytology turned negative	39	82.9
Histopathological tumor regression		
TRG 1a (complete)	3	7.1
TRG 1b	11	26.2
TRG 2	16	38.1
TRG 3	`12	28.6

### Survival

**Figure 1:**
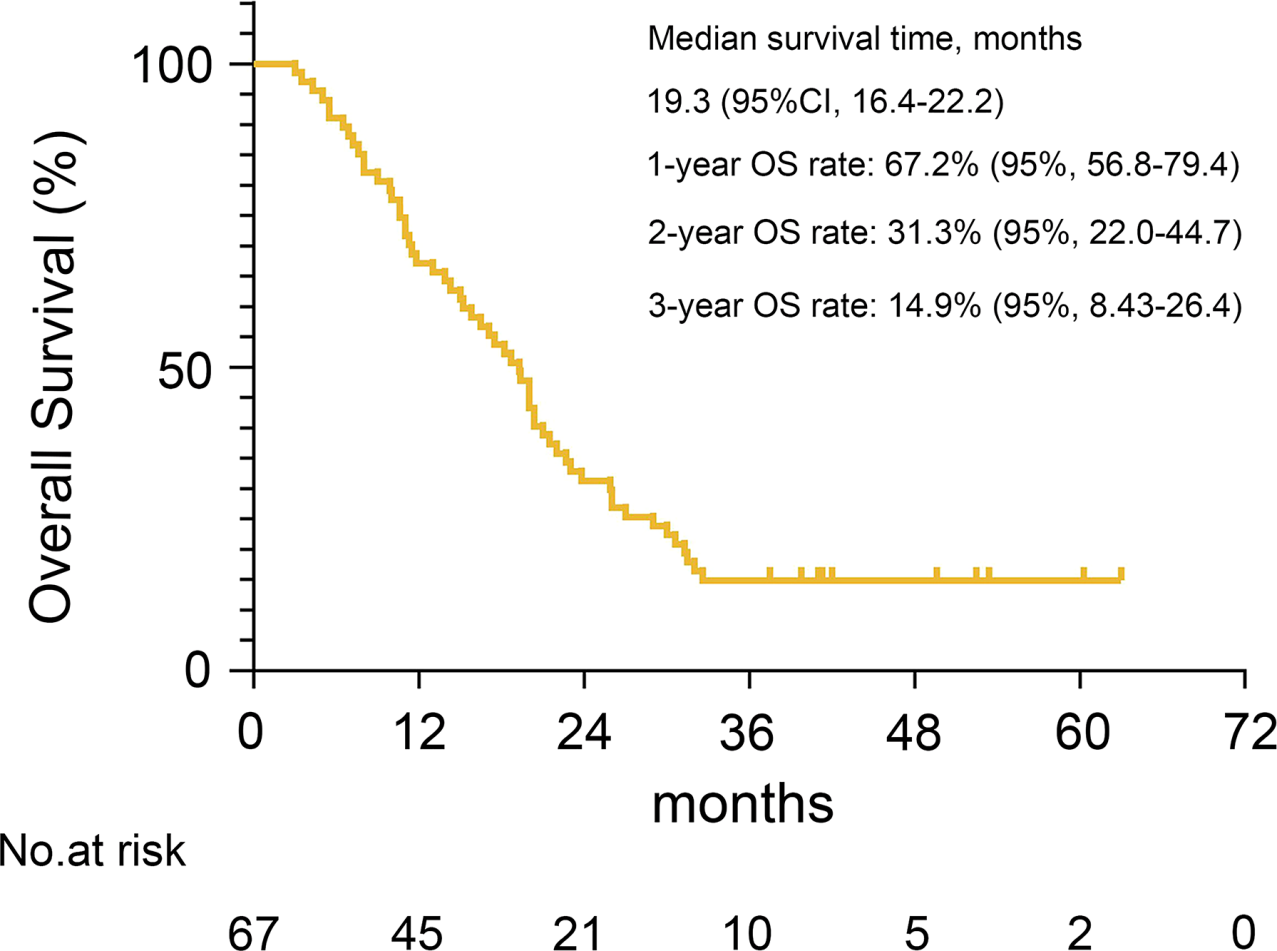
Kaplan-Meier plot is shown for the MST, 1-year, 2-year and 3-year OS rates with 95% confidence interval (CI).

**Figure 2:**
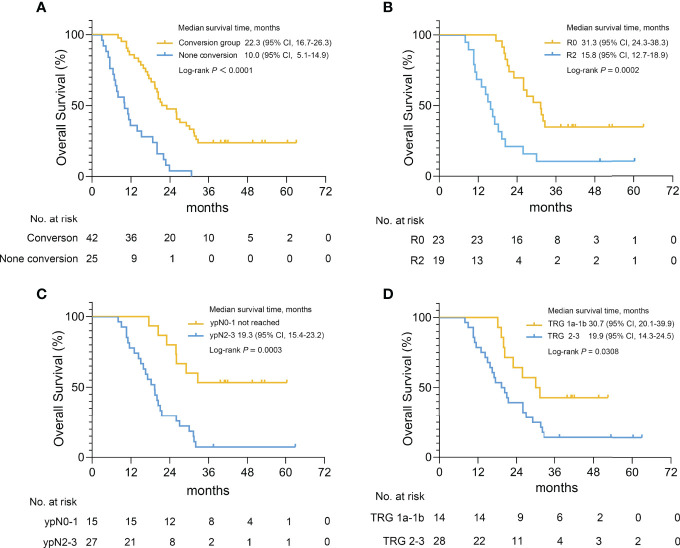
Survival curves for gastric cancer patients with peritoneal metastasis in different groups. **(A)** Kaplan-Meier plot for the MST of patients who underwent conversion surgery or not, for the MST of patient who underwent R0 and R2 resection **(B)**, for the MST of patients with ypN0-1 and ypN2-3 pathological nodal stages **(C)**, for the MST of patients with TRG 1a-1b and TRG 2-3 **(D)**.

The primary endpoint was met with 1-year, 2-year and 3-year OS rate was 67.2%, 31.3% and 14.9%, respectively (**Figure 1**), and the median survival time (MST) was 19.3 months (95% CI, 16.4-22.2 months) in all 67 patients. There was no statistical difference between patients with high and low PCI scores, although there was a trend that patients with higher PCI scores had poorer survival in terms of MST (PCI 0-9: 27 months, PCI 10-19: 18.7 months, PCI 20-39: 17.9 months, *P*=0.281; **Supplementary Figure S1**). However, the MST of patients who underwent conversion surgery was 22.3 months (95% CI, 16.7-26.3 months) versus 10.0 months (95% CI, 5.1-14.9 months) for patients who were unable to undergo conversion surgery (*P*<0.0001) (**Figure 2A**). According to the extent of resection, the MST of patients who underwent R0 resection was 31.3 months (95% CI, 24.3-38.3 months), while the MST of patients who underwent R2 resection was only 15.8 months (95% CI, 12.7-18.9 months) (**Figure 2B**). The pathological nodal stage also affected the survival of the patients, and the MST of patients with ypN0-1 was not reached versus 19.3 months (95% CI, 15.4-23.2 months) in patients with ypN2-3 (*P*=0.0003) (**Figure 2C**). According to the pathological response, the MST of patients with TRG 1 (1a and 1b) was longer than patients with TRG 2-3 [30.7 months (95% CI, 20.1-39.9 months) versus 19.9 months (95% CI, 14.3-24.5 months), *P*=0.0308] (**Figure 2D**).

Relapse was observed in 33 of the 42 patients who underwent conversion surgery, and the peritoneum was the main site of relapse. Single peritoneal relapse was found in 18 patients, and another 8 patients had multiple relapse sites. Other sites of relapse were the distal lymph nodes, ovary, anastomotic stoma, liver, bone, lung and pleura, and relapse mostly occurred in multiple sites. In total, among these 33 patients, 21 patients had single relapse sites, and 12 patients had more than one site of metastasis (**Supplementary Table S4**).

### Prognostic Factors

The prognostic factors that influence the OS of GC patients with peritoneal metastasis are still unclear. Therefore, univariate and multivariate regression analyses were performed before NIPS and after conversion surgery. Unexpectedly, there were no clinical factors that were significantly associated with the survival of patients in the whole group before NIPS (**Supplementary Table S5**). Further univariate and multivariate Cox regression analyses indicated that conversion surgery, sex, extent of resection, pathological nodal stage and TRG were independent prognostic factors for the survival of patients who underwent conversion surgery (**Table 5**).

**Table 5:** Univariate and multivariate analysis of prognostic factors in conversion surgery patients.

Variable	Univariate analysis			Multivariate analysis		
	HR	95% CI	*P* value	HR	95% CI	*P* value
Conversion surgery						
No	0.26	0.15-0.45	<0.001	0.27	0.11-0.69	0.006
Yes						
Sex						
Female	0.41	0.18-0.96	0.040	0.26	0.10-0.66	0.005
Male						
Age						
< 60	0.78	0.34-1.81	0.568			
≥ 60						
Conversion cycles						
< 6	0.86	0.43-1.73	0.681			
≥ 6						
Type of gastrectomy						
Distal	0.69	0.33-1.44	0.323			
Total						
Combination of resection						
Gall bladder	0.46	0.11-1.99	0.296			
Pancreatic body and tail	0.71	0.16-3.23	0.657			
Ovary	3.15	0.26-37.7	0.365			
Extent of resection						
R0	1.89	1.32-2.70	0.001	2.80	1.74-4.52	< 0.001
R2						
Lymphadenectomy						
D2	0.14	0.04-0.44	0.001	0.39	0.11-1.39	0.148
D1						
Pathological tumor stage (ypT)						
ypT0-2	4.17	1.45-12.00	0.008	1.89	0.56-6.34	0.302
ypT3-4						
Pathological nodal stage						
ypN0-1	4.28	1.82-10.09	0.001	3.37	1.27-8.99	0.015
ypN2-3						
TRG grade						
1	1.89	1.32-2.70	0.001	2.87	1.12-7.37	0.028
2-3						
Metastatic ovary resection						
No	0.58	0.18-1.87	0.366			
Yes						

## Discussion

Peritoneal metastasis is the most frequent type of metastasis and recurrence in patients with advanced gastric cancer (AGC), even after radical gastrectomy. It is reported that 10%-20% of patients are found to have peritoneal seeding at the time of potentially curative resection, especially in T3 or T4 tumor (19). Peritoneum is the only site of the first recurrence in 40%-60% in patients with AGC and is an independent cause of death in approximately 30-50% of patients with AGC (20). The strategy of applying NIPS followed by gastrectomy has been used as one of the optimal therapies for patients with peritoneal metastasis of AGC with encouraged results (21, 22).

Paclitaxel (PTX) is a chemotherapeutic agent with high molecular weight and lipid solubility (23), when administered intraperitoneally, PTX maintains a high concentration of the drug in the peritoneal cavity over a long period (24). Intraperitoneal infusion of PTX has been indicated to be an efficient method for treating ovarian cancer and GC with peritoneal metastasis and demonstrated a survival benefit in these patients (25). Recently, intraperitoneal chemotherapy became more popular because of the PHOENIX-GC clinical trial, although the primary endpoint OS failed to reach the predetermined level of significance (11), NIPS therapy was still suggested to be efficient in the treatment of GC with peritoneal metastasis (26). Conversion surgery after NIPS therapy contributed to the prolongation of survival, with postoperative MST of 12.8-43.2 months (27, 28). On the contrary, the MST of patients unable to undergo conversion surgery was 8.0-10.3 months (27, 29). Therefore, conversion surgery after NIPS therapy may be a proper option for GC patients with peritoneal metastasis.

Since GC is a highly heterogeneous tumor, which group of patients are more likely to benefit from NIPS treatment and what is the best time to perform conversion surgery remain to be explored. In our study, we investigated the safety and efficacy of NIPS and analyzed the prognostic factors for GC patients with peritoneal metastasis. To our knowledge, this is the first phase II study to evaluate NIPS in GC patients with peritoneal metastasis in China. The 3-year OS rate in our study was comparable with the results of PHOENIX trial; however, in PHOENIX phase II study, the 1-year and 2-year OS rates were 77.1% and 44.8%, respectively, compared with 67.1% and 31.3% in this study (17). The main reason for the difference between the two groups may be that the extent of peritoneal metastasis in our group was relatively serious, with P3 accounting for about 90%, and there were 9 patients (9/32, 28.1%) with PCI scores ≥ 20 in the PHOENIX group versus 30 patients (30/67, 44.8%) in our group. And another factor might be the high rate of R2 resection in our group. Therefore, the extent and severity of peritoneal metastasis remain one of the key factors in determining the therapeutic outcome.

Conversion surgery has been proven to improve the survival of patients with peritoneal metastasis (28). The median course of conversional chemotherapy was 6 courses in our group, and there was no discrepancy in OS between the < 6 courses and ≥ 6 courses (**Supplementary Figure S2**). It may be suggested that usually after 6 courses, the sensitivity and the outcome of peritoneal metastases to NIPS therapy can’t be further improved even with prolonged treatment time. However, more than 6 courses of chemotherapy were still recommended in patients with PCI ≥20, this may not improve the survival but can select appropriate cases for conversion surgery.

Regarding the extent of surgery, a previous study showed no significant difference in OS between R0 and R1/R2 gastrectomy (22). In the early stage of the study, we performed conversion surgery mainly by confirming the obvious shrinkage of peritoneal metastasis, which resulted in a high proportion of R2 resection. Unfortunately, the patients with R2 resection had a poor prognosis compared with those with R0 resection (15.8 versus 31.3 months in MST), based on the results of this study, we proposed that the indication of conversion surgery should be as follows: 1) disappearance of peritoneal metastasis by second-look laparoscopy; 2) negative peritoneal cytology; 3) no other distant metastasis; 4) downstaging of the primary tumor; 5) the patient’s general condition improved. Palliative resection should be avoided as far as possible in case with incomplete control of peritoneal metastases.

In terms of patients’ prognosis, pathological nodal stage was another independent factor, which has not been noticed previously. After conversion surgery, patients with ypN2 or ypN3 had a detrimental prognosis compared with patients with ypN0-1. An interesting finding was that the prognosis in male patients was better than female patients after conversion surgery (32.1 months versus 19.7 months in MST, *P*=0.033, **Supplementary Figure S3**). This finding provided a clue that male patients might have better survival once they proceed with conversion surgery. However, this phenomenon should be verified in the future phase 3 trial. We evaluated the pathological response of patients by TRG after conversion surgery. Patients with TRG 1 had a preferable prognosis compared with patients with TRG 2 and 3 (30.7 months versus 19.9 months in MST, *P*=0.038). These results suggested that regression and downstaging of the primary cancer and metastatic lymph nodes was as important as the elimination of the peritoneal metastases, so as to make opportunities for the implementation of radical conversion surgery.

Serum tumor markers have been identified to be correlated with the clinical statu`s of gastric cancer patients with peritoneal metastasis. Emoto et al. showed that serum CA125 and CA72-4 are clinically useful markers in diagnosis, evaluating the efficacy of chemotherapy, and predicting the prognosis of patients with peritoneal dissemination (30). The CEA mRNA levels were also reported to reflect the response of peritoneal metastases to induction intraperitoneal chemotherapy. In this trial, we have collected the tumor markers information of the enrolled patients and we will analyze these data in the future study (31).

Concerning safety, all patients received at least 3 courses of NIPS therapy. Hematologic and nonhematologic toxicities were tolerable and manageable during the treatment. Port-related complication incidence was more frequent in the early stage of the study due to the shortage of implantation experience. Even so, most of the complications could be controlled by conservative treatments and an optimized implantation procedure was performed afterwards (32). Although the prevalence of surgery-related complications was 42.8% (18/42), the most common complication was ileus (5/18), which was controlled by conservative treatments. Two patients had intra-abdominal bleeding and anastomotic leakage complications, which were managed with hemostatics, antibiotics and drainage. Other complications were seldom and well controlled by nonsurgical interventions.

However, many crucial issues have to be confirmed in the future study, such as the comparison of the NIPS with systemic chemotherapy in terms of the survival benefits, the criteria and appropriate timing for conversion surgery and the subsequent treatments. At present, our phase 3 randomized controlled trial on NIPS for GC with peritoneal metastasis is ongoing, which compares the efficacy of NIPS with that of systemic chemotherapy and will provide more evidence for resolving the encountered challenging problems (ChiCTR-IIR-16009802).

In conclusion, NIPS treatment appeared to be a safe, effective and well-tolerated therapeutic option for the treatment of GC patients with peritoneal metastasis. For the first time, we indicated that male patients, patients who underwent R0 resection, and patients with ypN0-1 or TRG 1 after conversion surgery are more likely to benefit from the NIPS therapy.

## Data Availability Statement

The original contributions presented in the study are included in the article/**Supplementary Material**. Further inquiries can be directed to the corresponding authors.

## Ethics Statement

The studies involving human participants were reviewed and approved by Ruijin Hospital Ethical Review Board. The patients/participants provided their written informed consent to participate in this study.

## Author Contributions

CY, MY and Z-GZ were responsible for the conception and design of the study. Z-YY, FY, SL and C-YH were responsible for patient recruitment, data acquisition, data analysis and writing of the manuscript. WX, J-WW, W-QX and MS collected the data and edited the manuscript. Z-QW, Z-TN, X-XY, Y-NZ, and Z-LZ collected the data and analyzed the data. W-TL, JZ, HZ and CL supervised and edited the manuscript. All authors have read and approved the final manuscript.

## Funding

This research was supported by from National Natural Science Foundation of China [grant number 81772518 and 81902393] and Multicenter Clinical Trial of Shanghai Jiao Tong University School of Medicine [grant number DLY201602].

## Conflict of Interest

The authors declare that the research was conducted in the absence of any commercial or financial relationships that could be construed as a potential conflict of interest.

## Publisher’s Note

All claims expressed in this article are solely those of the authors and do not necessarily represent those of their affiliated organizations, or those of the publisher, the editors and the reviewers. Any product that may be evaluated in this article, or claim that may be made by its manufacturer, is not guaranteed or endorsed by the publisher.
