# Supplementary material for: Efficacy and Safety of Conversion Therapy by Intraperitoneal and Intravenous Paclitaxel Plus Oral S-1 in Gastric Cancer Patients With Peritoneal Metastasis: A Prospective Phase II Study
Source: Front Oncol. 2022 Jun 20;12:905922. doi: 10.3389/fonc.2022.905922 (PMC9251062; doi:10.3389/fonc.2022.905922)
Supplement: Supplementary file 1 [file DataSheet_1.docx]

**Supplementary Figures and Tables**

**Efficacy and safety of conversion therapy by intraperitoneal and intravenous paclitaxel plus oral S-1 in gastric cancer patients with peritoneal metastasis: a prospective phase II study**

**Figure S1** Kaplan-Meier plot for the MST of patients with different PCI scores.

**Figure S2** Kaplan-Meier plot for the MST of patients received < 6 courses and ≥ 6 courses of NIPS.

**Figure S3** Kaplan-Meier plot for the MST of male and female patients before (A) and after conversion surgery (B).

**Table S1** Eligibility criteria for enrolling patients.

**Table S2** Port-related complications.

**Table S3** Surgery-related complications.

**Table S4** Relapsed patterns after conversion surgery.

**Table S5** Univariate analysis of prognostic factors before conversion surgery.

**Figure S1** Kaplan-Meier plot for the MST of patients with different PCI scores.

**
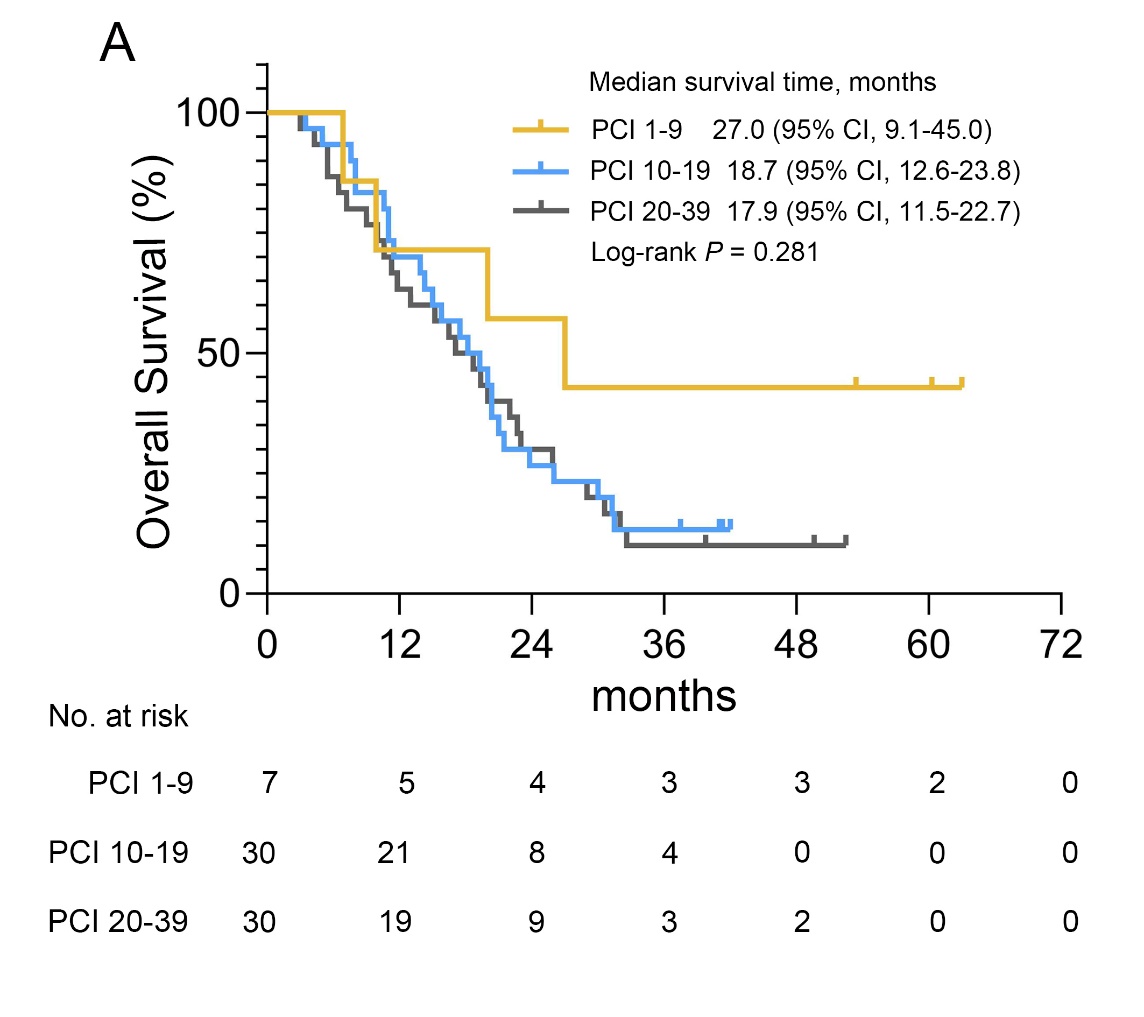
**

**Figure S2** Kaplan-Meier plot for the MST of patients received < 6 courses and ≥ 6 courses of NIPS.

**
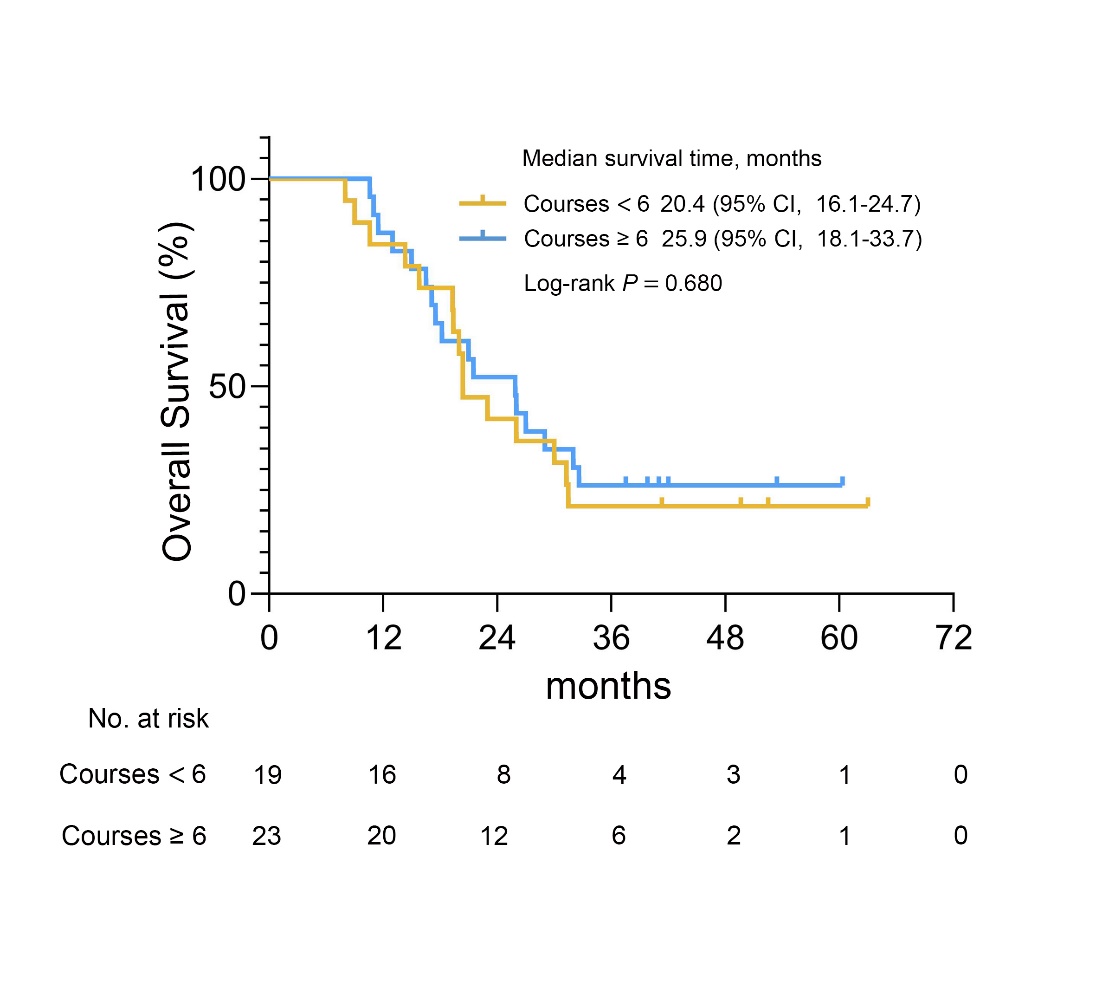
**

**Figure S3** Kaplan-Meier plot for the MST of male and female patients before (A) and after conversion surgery (B).

**
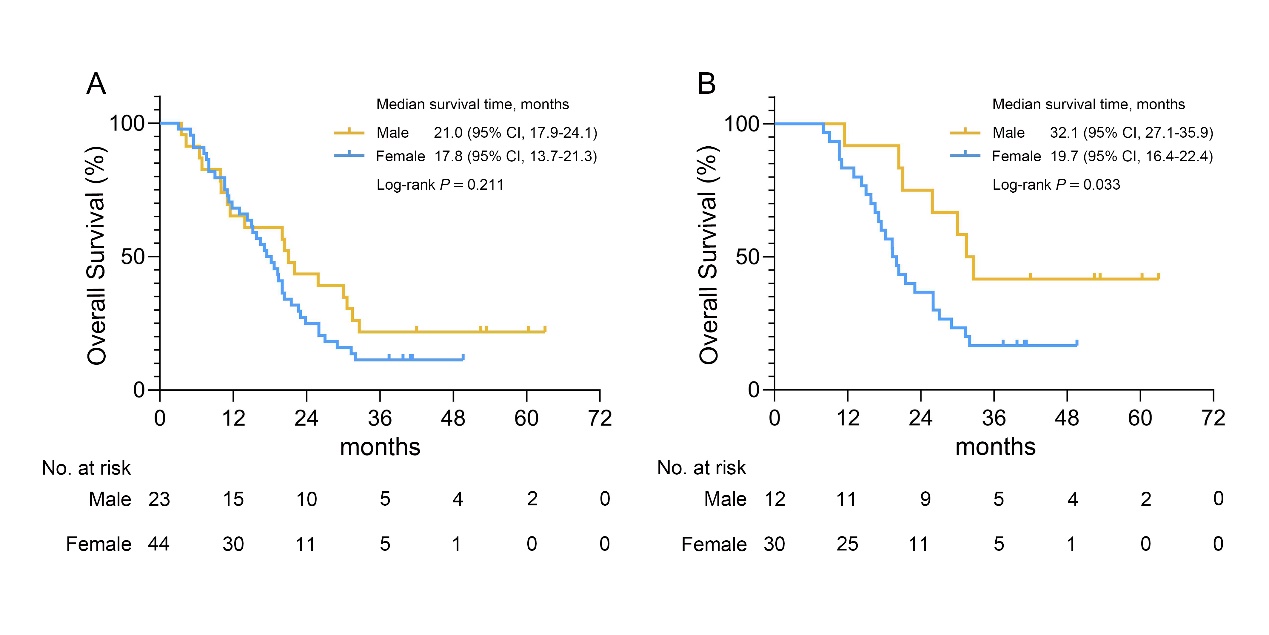
**

**Table S1** Eligibility criteria for enrolling patients.

| **Inclusion criteria** |
| --- |
| 1) Histologically confirmed gastric adenocarcinoma;  2) Peritoneal metastases from gastric cancer requiring definitive diagnosis by laparoscopy not just positive peritoneal cytology;  3) Patients without gastric outflow tract obstruction and intestinal obstruction;  4) No prior treatment with chemotherapy, radiation therapy, targeted therapy or immunotherapy;  5) Age between 18 and 75 years at registration;) Eastern Cooperative Oncology Group (ECOG) score ≤ 2;  6) Expected life expectancy ≥ 3 months;  7) Adequate bone marrow, liver, and renal functions. |
| **Exclusion criteria** |
| 1) Confirmed of evidence of distant metastasis other than peritoneal metastasis (e.g.liver metastasis, lung metastasis, para-aortic lymph node metastasis, etc.);  2) Pathologically detected with HER2 positive gastric cancer;  3) During pregnancy, within 28 days of post parturition, or during lactation;  4) Synchronous or metachronous (within 5 years) malignancies.  5) Severe mental disease, uncontrolled epilepsy, or central nervous system disease;  6) Clinically severe (i.e. active) heart disease, such as symptomatic coronary heart disease, New York Heart Association (NYHA) class II or more severe congestive heart failure or arrhythmia requiring drug intervention, or a history of myocardial infarction in the last 12 months;  7) Upper gastrointestinal obstruction or abnormal physiological function or malabsorption syndrome may affect S-1 absorbers;  8) Known peripheral neuropathy (> NCI-CTC AE 1). However, patients with only disappearance of deep tendon reflex (DTR) need not be excluded;  9) Patients on steroid or immunosuppressant treatment after organ transplant;  10) Patients with severe uncontrolled recurrent infections or other severe uncontrolled concomitant disease;  11) Moderate or severe renal damage [creatinine clearance ≤ 50 ml/min], or serum creatinine > upper limit of normal (ULN);  12) Known dihydropyrimidine dehydrogenase (DPD) deficiency;  13) Anaphylaxis to paclitaxel or any research drug ingredient. |

**Table S2** Port-related complications.

| **Complication** | **Grade 1** | **Grade 2** | **Grade 3** | **Grade 4** | **Total** |
| --- | --- | --- | --- | --- | --- |
| Infection | 1 | 1 | 1 | 1 | 4 |
| Port rotation | 2 | 1 |  |  | 3 |
| Wound dehiscence |  | 1 |  |  | 1 |
| Inflow obstruction |  |  |  |  | 0 |
| Liquid accumulation | 5 | 1 | 1 | 1 | 8 |
| Subcutaneous metastasis |  |  |  | 1 | 1 |

**Table S3** Surgery-related complications.

| **Complication** | **Grade 1** | **Grade 2** | **Grade 3** | **Grade 4** | **Total** |
| --- | --- | --- | --- | --- | --- |
| Intra-abdominal bleeding |  | 1 |  |  | 1 |
| Pancreatic fistula |  | 1 | 1 |  | 2 |
| Wound infection |  | 1 |  | 1 | 2 |
| Anastomotic leakage |  | 1 |  |  | 1 |
| Ileus | 1 | 3 | 1 |  | 5 |
| Abdominal infection | 1 |  |  |  | 1 |
| Pulmonary infection |  | 1 |  |  | 1 |
| Urinary tract infection |  | 1 | 1 |  | 2 |
| Sepsis |  | 1 |  |  | 1 |
| Stenocardia |  | 1 |  |  | 1 |
| Perineum edema | 1 |  |  |  | 1 |
| Death |  |  |  |  | 0 |

**Table S4** Relapsed patterns after conversion surgery.

| Patterns | No. | Detailed components |
| --- | --- | --- |
| Peritoneal | 26 | 18 with single PM and 8 with multiple sites |
| Ovary | 2 | 2 combined with PM |
| Pleura | 2 | 1 combined with PM and 1 combined with DLN |
| Distal lymph nodes | 8 | 2 with single DLN and 6 with multiple sites |
| Anastomotic stoma | 1 | 1 combined with DLN |
| Hematogenous |  |  |
| Liver | 4 | 2 combined with PM and DLN, 1 combined with bone and DLN and 1 combined with PM and lung |
| Bone | 2 | 1 with single bone, 1 combined with liver and DLN |
| Lung | 3 | 1 combined with PM, 1 combined with DLN and 1 combined with PM and liver |

**Table S5** Univariate analysis of prognostic factors before conversion surgery.

| **Variable** | **n** | **HR** | **95% CI** | ***P*** |
| --- | --- | --- | --- | --- |
| Sex |  |  |  |  |
| Female | 23 | 0.70 | 0.40-1.23 | .22 |
| Male | 44 |  |  |  |
| Age |  |  |  |  |
| < 60 | 51 | 0.84 | 0.45-1.56 | .58 |
| ≥ 60 | 16 |  |  |  |
| BMI |  |  |  |  |
| < 23 | 44 | 0.89 | 0.51-1.54 | .67 |
| ≥ 23 | 23 |  |  |  |
| ECOG PS |  |  |  |  |
| 0 | 27 |  |  |  |
| 1 | 31 | 0.48 | 0.21-1.09 | .08 |
| 2 | 9 | 0.76 | 0.35-1.67 | .50 |
| Peritoneal metastasis |  |  |  |  |
| P1 | 2 |  |  |  |
| P2 | 5 | 0.01 | 0.01-7.98 | .97 |
| P3 | 60 | 1.65 | 0.66-4.17 | .29 |
| Amount of ascites |  |  |  |  |
| None | 9 |  |  |  |
| Small | 26 | 0.60 | 0.26-1.38 | .23 |
| Moderate | 32 | 0.77 | 0.44-1.35 | .35 |
| Histologic type |  |  |  |  |
| Adenocarcinoma | 53 |  |  |  |
| Mucinous cell | 3 | 0.99 | 0.49-2.04 | .99 |
| Signet ring cell | 11 | 1.44 | 0.39-5.38 | .58 |
| Pathological grading |  |  |  |  |
| Moderately | 2 |  |  |  |
| Poorly | 42 | 1.09 | 0.25-4.67 | .91 |
| Unknown | 23 | 0.61 | 0.35-1.06 | .08 |
| PCI score |  |  |  |  |
| 0-9 | 7 |  |  |  |
| 10-19 | 30 | 0.44 | 0.15-1.25 | .12 |
| 20-39 | 30 | 0.93 | 0.54-1.59 | .79 |
| Ovarian metastasis (Female) |  |  |  |  |
| With | 26 | 1.31 | 0.68-2.50 | .42 |
| Without | 18 |  |  |  |
